# Familismo, Lesbophobia, and Religious Beliefs in the Life Course Narratives of Chilean Lesbian Mothers

**DOI:** 10.3389/fpsyg.2020.516471

**Published:** 2020-10-20

**Authors:** Victor Figueroa, Fiona Tasker

**Affiliations:** ^1^Facultad de Humanidades y Ciencias Sociales, Universidad UNIACC, Santiago, Chile; ^2^Department of Psychological Sciences, Birkbeck, University of London, London, United Kingdom

**Keywords:** lesbian, mothers, Chile, familismo, familism, religiosity, lesbophobia

## Abstract

This study aimed to explore the life course identity projects of Chilean lesbian mothers who conceived their children within the context of a previous heterosexual relationship. By exploring the case of Chile, this study examined the family lives of lesbian mothers within the context of a Latino heteronormative society with a Christian (mainly Catholic) heritage. Individual interviews were conducted with eight participants aged between 27 and 40 years old (mean age of 33 years) who were recruited through snowballing and social media. A Structural Narrative Analysis of participants’ stories was conducted within a Life Course Perspective theoretical framework. The study found that participants initially followed a heterosexual path to conform to their family of origin and social expectations. After building their own heterosexual family projects and having their children with a man, most participants felt pressured to continue within a heterosexual path and postponed their transition to a lesbian identity trajectory despite a growing feeling that a lesbian identity would be personally fullfilling. Although participants felt proud of their identities, they struggled to express their same-gender feelings because lesbians were often seen as inappropriate models for children within Chilean society. Crucially, lesbian mothers continued to be able to count upon support for their parenting from their own mother despite intense disapproval from their family of origin and often continued opposition from ex-husbands/partners. The findings of this study revealed the strong impact of familismo, lesbophobia and Christian religious beliefs on the life course experiences of Chilean lesbian mothers. Implications for therapy and counselling with lesbian mothers living in Latino countries are reviewed.

## Introduction

The study of the family life of lesbian mothers (LM) has been well documented within developmental psychology (Tasker and Patterson, 2007; Patterson and Riskind, 2010). Notwithstanding, most research has been conducted in Western European and English-speaking countries (Golombok, 2015). Little is known about the challenges facing by LM in other socio-cultural contexts (Lubbe, 2013). In recent years, there has been growing interest in the study of the family life of LM living in Latino countries (Sánchez et al., 2004; Pinheiro, 2006; Santos and Alves de Toledo, 2006; Libson, 2012; Palma et al., 2012; Uribe, 2014). These studies have revealed that lesbophobia and the legal/political context of Latino societies have played a crucial role in the ways in which LM experience their family life and navigate within the public domain. For example, studies conducted in Argentina, Brazil, Chile, and Mexico have indicated that some LM initially embark upon heterosexual relationships to conform to their family of origin’s demands. Further, after defining as a lesbian, they often hide their sexual identity in the public domain and usually restrict disclosure to within the family context. Besides, when LM establish an intimate partnership they also begin to feel concerned because of the lack of socio-legal protection to respect their status as a couple (Sánchez et al., 2004; Pinheiro, 2006; Santos and Alves de Toledo, 2006; Herrera, 2009; Jara and Araujo, 2011; Palma et al., 2012).

Nevertheless, following the postcolonial framework, we pay attention to the complexity of examining cultural diversity (Coronil, 2015). While LM living in Latino societies share similar experiences (such as contending with strong lesbophobic element common to Latino culture) differences among countries can be identified. For example, previous studies have shown that some Brazilian LM live with the fear of being attacked because of the context of violence (in all its forms) and hate crimes against sexual minorities in which they live (Pinheiro, 2006; Santos and Alves de Toledo, 2006). Likewise, other studies have indicated that some Mexican LM taught their children not to express non-heterosexual behaviours, such as kissing another child of the same gender, to avoid homophobic reactions (Haces, 2006). In a parallel fashion previous studies focused on Chilean LM have indicated that lesbian women feared potentially losing custody of their children, being threatened by their heterosexual ex-partners, or even by their family of origin, during disputes related to separation or divorce (Herrera, 2009; Jara and Araujo, 2011).

Despite the insightful understanding of the family life of LM living in Latino countries previous studies have shown, the role of Christian religious beliefs and values on the family experiences of LM have received less attention. A study conducted with Catholic Hispanic LM in the United States found that religion produced an identity conflict given its potential incompatibility with the role of a “good mother” (Tuthill, 2016). Thus, it seems that Christian religious beliefs and values might have an important impact on the family life of LM within a Latino cultural context, even for the LM in Tuthill’s study who were all Latina women living in the United States. The present study was conducted with LM who, at the time the study, were all living in Chile, a Latino country with a strongly Christian (mainly Catholic) religious heritage. For example, a study that explored discourses concerning lesbian and gay (LG) parenting in Chile indicated that some Christian heterosexual women expressed the view point that having LG parents could disrupt a child’s gender and sexual identity development while other heterosexual women suggested ‘no we’re not culturally ready for that yet’ (Figueroa and Tasker, 2019).

Furthermore, studies conducted with Latina lesbians (LL) in the United States have revealed that LL’s parents holding Catholic religious beliefs often portrayed homosexuality as a sin and thus an undesirable sexual orientation. For example, Acosta (2010) reported that LL in the United States often stated that their mothers used religion to protect their daughters from the sin of homosexuality by sending their daughter to talk to the priest. Another study conducted with lesbians in Chile found that family members’ negative views of homosexuality based on Catholic beliefs influenced participants’ self-rejection (Herrera, 2007).

In contrast, studies have shown that Latino families do not necessarily expel their lesbian daughter from the family circle (Espín, 1987; Herrera, 2007; Asencio, 2009; Acosta, 2010). Thus, in spite of a family’s rejection of lesbianism, LL often receive support from their families after disclosure. This particular support has been described as an expression of Latino familism which implies a strong interdependence observed within Latino families (Muñoz-Laboy, 2008). Thus, Latino families make major efforts to preserve familial bonds by avoiding confrontation regardless of family heteronormative expectations (Acosta, 2010). Heteronormativity has been described as an ideology that promotes heterosexuality as the norm (Oswald et al., 2005; Nardi et al., 2013).

### The Chilean Sociolegal Context

The homo/lesbophobic context existing in Chile, as in the rest of Latin America, has been largely associated with the historical rejection of homosexuality fostered by the Catholic Church (Akerlund and Cheung, 2000; Bozon et al., 2009). However, the Catholic Church has been seen to have a greater impact on law and policy in Chile than in other Latin American countries (Htun, 2003). Indeed, same-sex marriage and adoption by same-sex couples have been legalised in Argentina, Brazil, Colombia and Uruguay and recently in 2019 in Ecuador too. In contrast, in Chile, neither same-sex marriage nor adoption have not been legalised yet and there is a slow movement in favour of same-sex couples when compared to other South American countries.

Regarding Chilean legislation, in recent years important legal changes have been enacted in favour of LGBTQ people. These legal changes have included the decriminalisation of homosexuality (1999), the Law Against Discrimination (2012), the Civil Union for other- and same-sex couples (2015)^1^, and the Gender Identity Law (2018). Furthermore, in Peña (2017) the Supreme Court of Justice granted a gay father and his male partner, the custody of their two children. In addition, recently in 2020, a Chilean Family Court recognised two women as the legal mothers of a child conceived through reproductive technologies. These rulings are very different from what happened in the case of Judge Karen Atala, who had her daughters removed from her custody by the Supreme Court of Justice in 2004 because the court considered that living with a lesbian couple was a risk to the girls’ psychosexual development.

Despite the restrictive Chilean legal context toward LG parenting, Chilean people’s approval of homosexuality seems to be at similar levels to that in other Latino countries with less restrictive legislation toward same-sex couples. For example, one international survey found that 74% of Argentineans, 68% of Chileans, 61% of Mexicans, and 60% of Brazilians agreed that society should accept homosexuality (Pew Research Center, 2013). Another international survey found similar mean levels of approval of homosexuality in Argentina (5.6), Puerto Rico (5.6), Chile (5.0), and Brazil (5.0), in a scale ranging from “Never justifiable” (1) to “Always justifiable” (10) (World Values Survey, 2020).

Concerning public approval of same-gender parenting in Chile, a national survey of 1303 people revealed that 44% of the participants agreed or strongly agreed that “a couple of women (lesbians) can raise a child as well as a heterosexual couple can” (Instituto de Investigación en Ciencias Sociales, 2015). While 40% of the participants agreed or strongly agreed that “a couple of men (gays) can raise a child as well as a heterosexual couple can.” These data reveal that social approval particularly of same-sex parenting was still low in Chile despite that 63% of the participants in the same study agreeing or strongly agreeing “homosexuality is a sexual option as valid as any other.” The national survey conducted by Cadem (2018) revealed similar results: while only 44% of Chileans approved of adoption by same-sex couples, 60% approved of same-sex marriage.

Another Chilean national survey has indicated that religious affiliation might have an impact on people’s prejudice toward LG parenting. The study showed that 41% of participants who identified as Catholic, and 23% of those who identified as Evangelical, agreed with the right of homosexual couples to adopt children (Instituto de Investigación en Ciencias Sociales, 2014). Chilean people still report a high level of religiosity: 80% of people reported believing in God (Centro de Estudios Públicos, 2018) although the same survey indicating that Catholic Faith had declined from 73% (1998) to 55% (2018), while Evangelism had slightly increased from 14% to 16% during the same period.

The Chilean sociolegal context has been particularly complex for lesbians. “Homosexuality,” including “lesbianism,” has historically been portrayed as a sin and a transgression of moral norms (Contardo, 2011). Furthermore, although gay movements reached visibility during the 90s, lesbian women’s voices were less visible than gay men’s demands during this period. However, by the beginning of the 21st century, new lesbian-led movements emerged, such as “*Rompiendo el Silencio*” (Breaking the silence), giving visibility to lesbian voices. Further factors important in promoting Chilean social awareness and acceptance of lesbianism were the high-profile recognition of Nobel prize winner Gabriela Mistral’s relationship with Doris Palma and also the media coverage of Judge Karen Atala’s successful custody battle through the courts. Nevertheless, many Chilean people still see lesbianism as incompatible with motherhood and consider that it is not normal for children to be growing up in a lesbian-led household (Herrera, 2009; Figueroa and Tasker, 2019).

### Research Aims

This contrasting context - continued legal restriction versus comparative public acceptance - seems to be interesting when exploring the family life of LM, especially, when we consider that previous studies have indicated that lesbians and gay men in Chile still experience high levels stigmatisation and internalised homo/lesbophobia (Figueroa and Tasker, 2014; Cárdenas et al., 2018). Internalised homophobia has been defined as “the gay person’s direction of negative social attitudes toward the self, leading to a devaluation of the self and resultant internal conflicts and poor self-regard” (Meyer and Dean, 1998, p. 161). We used the term internalised lesbophobia when lesbians experienced negative feelings toward self (Guthrie, 2005; Fogaça et al., 2011). Just as Latinos live with machismo, so Latinas live with the negative emotional cognitions and sociocultural restrictions of Marianismo (Nuñez et al., 2016). We suggest that internalised homo/lesbophobia instilled by traditional Latino family values based in Christianity - particularly Catholicism - often keep sexual minority people from living more visible lives and that this is especially the case when they embody a direct challenge to a central figure in the Catholic family – the mother.

We employed Life Course Perspective as our main theoretical framework for the present study of Chilean LM since this helps to locate lesbian parented families both within a wider socio-cultural and historical context and locates an individual life within a developmental and family framework (Elder, 1998; Cohler, 2005). Relying on a life course perspective, sexual identity development could be understood as a process of narrative engagement throughout which individuals actively make sense of their same-gender desire in a particular historical and cultural context (Hammack and Cohler, 2009). Thus, we also used a narrative approach to analyse how these group of LM we interviewed made sense of their same-gender desire through narratives available to them as they grew into womanhood and as mothers within a Chilean cultural context.

Narratives can be understood as stories people tell about their own lives which are influenced by cultural conventions, language usage and historical circumstances (Bruner, 1987). According to Murray (2008), people define themselves through narratives that bring a sense of order and temporal continuity to events. Hammack (2008) has highlighted how the cultural psychology of sexual identity development can be enriched by employing a narrative approach. From this standpoint, personal narratives are constructed and re-constructed throughout the life course, and are embedded in social interaction and social practice (Hammack, 2008).

In particular, our study explored the life course experiences of a group of Chilean LM who conceived their children through a previous heterosexual relationship, a situation relevant in countries with or without legislations enabling same-gender couples to access adoption or assisted reproductive technology services (Tasker and Rensten, 2019). Our study also examined how sexual identity and motherhood were negotiated in the private and public domains.

Given this purpose, our research questions were: How does Chilean LM coming from a previous heterosexual relationship develop their sexual identities over their life courses? What are the particular ways in which Chilean LM negotiate their identities as mothers and lesbians with their family of origin? How does Chilean LM negotiate their identities and their children’s identities in mainstream society? How do Christian religious beliefs and values shape the life course experiences of LM in Chile?

## Materials and Methods

### Participants

Our initial screening sample in this study was 16 Chilean LM from different Chilean cities. Criteria for participants’ inclusion were being a Chilean woman aged over 18 years old, currently identifying as lesbian, and being a mother of at least one child of any age. As this was an exploratory study due to scarcity of knowledge about Chilean LM, no other criteria were imposed by the initial sampling framework. Interviewees were aged between 27 and 56 years old, with a mean age of 37 years. After finishing data collection, the sixteen interviews were audio-analysed and six macro-narratives (overarching life stories) were identified across cases. Based on participants’ demographics features and the more represented macro-narratives across cases, eight participants were selected for the purpose of systematically exploring self-identity construction and experience within a homogeneous sample. We aimed to conduct a detailed case-by-case analysis with a small and homogenous sample. Thus, we did not consider saturation to close data collection (Smith, 2008). The selected sample for the current study were eight self-identified lesbian women who had conceived and given birth to their first child within the context of a heterosexual relationship, had been involved in at least one lesbian couple relationship, and were currently parenting their biological children or adolescents who were living with them at the time of the interview or who had previously done so. Thus, from the initial screening sample, three LM who had conceived and given birth their first child in the context of a lesbian couple relationship, two LM who had adult offspring, and one self-identified lesbian mother who had never been involved in a lesbian relationship were excluded from the analysis. The eight selected participants were aged between 27 and 40 years old, with an average age of 33 years. Four participants had divorced their husband, and three had ended a cohabitation/relationship with their child’s father. The last participant was a married woman who was living in a couple relationship with her husband at the time of her interview but who was actively considering leaving this relationship.

Seven participants identified as middle social class and one of a higher social class. The average family income was 1,037,500 CLP (1,348.75 USD) per month, ranging from 500,000 to 1,500,000 CLP. All eight participants were in paid occupations. One participant was finishing an MSc degree, and four had completed undergraduate studies. Another two participants had begun undergraduate studies but not yet completed them at the time of the study. The last participant had completed secondary education. Thus, the sample as a whole was relatively middle class and educated compared to Chilean national data.

Six participants lived in Santiago. One interviewee lived in Talcahuano, and another participant lived in Rancagua. Four participants did not participate in any religious activity or hold religious beliefs, two identified as Catholic, one as Christian, and one reported believing in God but holding no denominational allegiance. A summary of each participant’s details and the pseudonyms given to participants are listed in Table 1.

**TABLE 1:** Demographic information for participants.

Participant	Age	Education	Marital/Relationship Status	Socio-economic Level	Childhood Religion/Adult Religion
Teresa	36	Secondary school completed	Divorced	Middle	Catholic/No
Camila	29	MSc student	Separated	Middle	Catholic/No
Julia	35	Graduated	Divorced	High	Catholic/No
Carla	31	Graduated	Separated	Middle	Catholic/Belief in God
Paula	38	Graduated	Divorced	Middle	Catholic/No
Jimena	27	Undergraduate student	Separated	Middle	Not reported/Catholic
Marcela	32	Undergraduate student	Married	Middle	Christian/Catholic
Beatriz	40	Graduated	Divorced	Middle	Catholic/Catholic

All eight participants were biological mothers. The mean age for the first pregnancy was 23.5 years, ranging from 22 to 30 years. Participants’ children were seven girls and five boys, with a mean age of 10 years old, ranging from 4 to 16 years. All participants’ children were enrolled in primary or secondary education as expected according to their chronological ages. Seven participants were living with their children (see Table 2 containing participants’ children’s details).

**TABLE 2:** Participants’ children’s details.

Participant	Sex	Age	Educational level	Religion	Living with	Since
Teresa	M	13	Primary, 8th year	No	Father	7 months
	M	10	Primary, 4th year	No	Father	7 months
Camila	F	7	Primary, 2nd year	No	Mother	Birth
Julia	F	6	Pre-School	Catholic	Mother	Birth
Carla	F	9	Primary, 3rd year	No	Mother	Birth
Paula	M	16	Secondary, 1st year	Catholic	Mother	Birth
	F	13	Primary, 7th year	Catholic	Mother	Birth
	M	9	Primary, 4th year	Catholic	Mother	Birth
Jimena	M	4	Pre-School	No	Mother	Birth
Marcela	F	10	Primary, 6th year	Catholic	Mother Father	Birth
Beatriz	F	15	Secondary, 2nd year	Buddhist	Mother	Birth
	F	10	Primary, 5th year	Catholic	Mother	Birth

Only one participant was not living with her children at the time of the study. All participants’ children were in contact with their biological father and received support from him, whether through shared childrearing, economic support, or sharing time together. Seven participants were involved in a lesbian couple relationship at the time of the study. But only three women were cohabitating with their lesbian partner when interviewed.

#### Recruitment

Recruitment of volunteers for this study was initially conducted through collaboration with two widely known Chilean sexual minority organisations based in Santiago, the ‘Movement of Homosexual Integration and Liberation’ (MOVILH) and Equal Foundation (Iguales). Invitations for the present study were displayed at the premises of both MOVILH and Iguales and publicised through each organisation’s internet network. In addition, a Facebook page was created for the study by the first author and invitations to participate were periodically published on this page. Five participants contacted the first author via Facebook and three via email. All interviewees were volunteers and none were paid for their participation.

### Interview

A semi-structured interview was designed for the study’s purpose (Gergen, 2010). Open-ended questions were constructed in advance and further prompts requests for clarification or expansion were requested during the interviews. Participants also were given the possibility at the end of the interview of raising other issues they thought relevant. The interview schedule began with an open question inviting participants to narrate their own life story about how they started to identify as a lesbian mother, a narrative life course interview opening question similar to the one suggested by Murray (2008). Further specific questions were constructed in advance as prompts in the case participants required a guide to address relevant topics according to the study’s purpose. Examples of these questions were the following: Had you thought about becoming a parent before you actually did? How did your parenting come about? When did you first become aware or begin to define yourself as a lesbian? Has this definition changed over time? Have you told other people about you being lesbian? How do you manage your motherhood and your lesbianism in your everyday life? How do your mother and lesbian identities fit in with other areas such as your work, children’s school, extended family, friends? In this way, the interviewer (first author) invited but did not focus attention solely on the role of family members in LM lives but instead opened questions areas. Encouraging participants to tell their own stories in this way empowered them to highlight the features of their lives that mattered to them as opposed to simply referring to the interview focus (Riessman, 2008; Hollway and Jefferson, 2013).

#### Interview Procedure

After participants contacted the researcher to express their interest in taking part in the study, they were given further information on the study and a choice of venues for the interview. Participants were given the interview schedule, alongside additional information on the study to know in advance that the topic would be addressed via a life story style interview. Thus, participants had probably reflected to a greater or lesser extent on what they were going to say in advance of the interview, empowering participants as they organised and prepared to present their stories.

Interviews were conducted between September 2013 and January 2014. Face to face interviews were conducted with each participant in different locations of their choosing. Five interviews were carried out in different cafes, one at MOVILH premises, one at a participant’s workplace, and another at a participant’s home. Of these interviews, seven were conducted in Santiago and one in Talcahuano. All interviews were conducted in Spanish by the first author and each lasted between 40 and 70 min. With each participant’s consent, interviews were audio-recorded.

Participants’ questions were answered over email, telephone and before interview commencement. Participants were informed that they could withdraw their consent to participate in the study at any time up to their final consent to include the checked transcript in the data set. Verbatim transcripts were encoded, and all participants’ information and study’s data were password protected and stored in the authors’ personal files. Recordings were erased after transcriptions were completed. This study was approved by the ethics committee of the Institutional Review Board at the host university.

Verbatim transcripts were made in Spanish by the first author. Personal information was disguised in the transcripts and pseudonyms were assigned to ensure the confidentiality of participants. Other names or potentially identifying details mentioned by participants were also changed. Each participant was given access to their own transcript and was given the opportunity to withdraw it or to make any changes or comments over a 2-month period. Only minor details were changed or clarified and none of the participants withdrew from the study.

#### Ethical Considerations

Interview questions directly explored participants’ personal stories of sexual identity and motherhood experiences. Thus, participants’ emotional states were observed during the interview in order to stop if necessary. We also planned to provide a back-up preliminary psychological support if required. After the preliminary session, participants could then be referred on to MOVILH’s support services for psychosocial counselling by one of two female psychologists, however no participants needed post-interview support.

### Narrative Analysis

Narrative analysis is a procedure that has enabled social scientists to analyse and interpret personal stories through which people make sense of their lived experiences (Riessman, 2008). In particular, the structural narrative analysis (SNA) focuses on narrative content, but with specific attention given to the narrative form, or how stories are told and organised by individuals. We followed Labov’s model, which has drawn particular attention to the elements of a narrative’s structure (Labov, 1972; Riessman, 2008). According to Labov (1972, p. 361), the “skeleton” of a narrative consists of a series ordered clauses which he called “narrative clauses.” Namely, Labov (1972) identified six narrative elements to guide the structural analysis: Abstract (What was this about?), Orientation (Who, what, where?), Complicating action (then what happened?), Evaluation (so what?), Result (What finally happened?), and Coda (which returns the listener to present). These six elements are summarised in Table 3 below, although not all six elements in order are necessary for a partial story to be narrated.

**TABLE 3:** Labovian narrative analysis list of structural codes used.

Codes	Elements (Labov, 1972)
AB	Abstract
OR	Orientation
CA	Complicating action
EV	Evaluation
RE	Result
CD	Coda

#### Structural Narrative Analysis Procedure

Relying on Riessman’s (2008) propositions for conducting structural narrative analysis, the following steps were addressed in each transcript analysed. Firstly, each transcript was re-read several times in order to identify each participant’s smaller personal stories which they used to illustrate their personal development: these episodes constituted individual mini-stories. Secondly, narrative clauses in the mini stories were thematically grouped, and emerging micro-narratives were constructed. Micro-narratives were later grouped into an overall life story (the macro-narrative). Thirdly, Labov’s (1972) structural elements were identified after a detailed analysis of each micro-narrative’s clauses. Fourthly, micro- and macro-narrative were re-organised into a life-course progression. The whole process was conducted individually with each participant’s transcript following the idiographic case centred approach, as suggested by Riessman (2010). After completing each participant’s final Labovian narrative, patterns of stories across cases were identified. Also, following Murray’s (2008) suggestions for the chronological organisation of narrative accounts, we identified the beginning, the middle, and the end in each participant’s macro narrative summary.

## Results

The themes that emerged from the structural narrative analysis reflected the coming out process of this group of Chilean LM from their early cognizance of their attraction to women until their adult years. Four main themes emerged from the analysis of participants’ narratives: (1) Conforming with the expected heterosexual path; (2) Experiencing a lesbian desire that needs to be expressed; (3) Conveying sexual identity to family of origin, friends and the child(ren)’s father; and (4) Conveying maternal sexual identity to the children. The first two themes focused on the processes through which participants developed their own understanding of their same-gender desire either when they were a childless woman or when they became mothers. The other two themes provided information about participants’ coming out process within private and public domains. Table 4 below contains the themes and sub-themes originated from the SNA.

**TABLE 4:** Themes and subthemes.

	Themes and Sub-themes
**(1)**	**Conforming with the expected heterosexual path**
(1.1)	*Lesbianism not expressed or selected as a life course project*
(1.2)	*Building a relationship and a family with a man*
**(2)**	**Experiencing a lesbian desire that needs to be expressed**
(2.1)	*Rethinking lesbianism as a life course identity project*
(2.2)	*Questioning the heterosexual family life project*
**(3)**	**Conveying sexual identity to family of origin, friends and the child(ren)’s father**
(3.1)	*Negotiating lesbian identity with family of origin*
(3.2)	*Negotiating lesbian identity with friends*
(3.3)	*Negotiating lesbian identity with the child(ren)’s father*
**(4)**	**Conveying maternal sexual identity to the children**
(4.1)	*Avoiding the disclosure of lesbian relationships to the children*
	Presenting lesbian partner as friend
	Hiding lesbian affectionate expressions
(4.2)	*Preparing the child for coming out as a lesbian mother*
	Teaching children to be tolerant
	(Planning) disclosure to the children

### Conforming With the Expected Heterosexual Path

All eight participants talked about their experiences of conforming to a heterosexual path during an initial period of their sexual identity life course. This theme split into two sub-themes: “*Lesbianism not expressed or selected as a life course project*” and “*Building a relationship and a family with a man.*”

#### Lesbianism Not Expressed or Selected as a Life Course Project

In spite of participants differences in the timing of their sexual identity life course, what characterised the accounts of all participants was that their lesbianism was not expressed or selected as life course plan during an initial stage of their sexual identity development. Some participants did not recognise lesbianism as a possibility for themselves (Camila, Julia and Jimena), while others (Carla, Marcela, and Beatriz) tried to hide their feelings because they feared the consequences of being seen as a lesbian because of their family pressures. Nevertheless, Teresa and Paula did consider lesbianism as an option for themselves during this first developmental period, but both opted for a heterosexual pathway at this point.

The three participants who tried to hide their same-gender feelings had assessed the negative consequences of being seen as a lesbian, mainly by their parents. Carla described the time when her mother realised about Carla’s lesbianism when Carla was 16 years old. In Carla’s evaluation below she decided she did care about her mother’s reaction and decided to deny her lesbian feelings. Carla concealed her lesbian desire to avoid her mother’s “suffering” (“sufrimiento” in Spanish), which seemed to be a strong family pressure for her. The word “suffering” used by Carla implied that lesbianism was a heavily undesired sexual expression within Chilean society when she was a youth:

**Table:** 

Lab	Clause
EV	*Carla: “so I said myself ’here I have two options*,
EV	*or I declare openly myself as lesbian and I see the suffering, because I saw the suffering of my mom*,
EV	*or I say no, that was a teenage foolishness which will pass’*,
RE	*and that was what I did, we are talking about 15 years ago when this*
CD	*[being lesbian] was even worse [within Chilean society]” (Narrative [N]1, Episode [EP] 1, Lines [L]166:170)*

Similarly, Marcela, who was from the southern and more traditional city of Talcahuano, narrated a short episode about her adolescent years when she decided to take a heterosexual path in trying to avoid any negative social consequences. Marcela heard her father talking negatively about if he had a gay or lesbian child, which then led Marcela to hide her early attraction to women. With her repetition of words Marcela also implied that she balanced hiding herself by emphasising being straight to herself and to her father and others. It seems that Marcela feared being “punished” by her “violent” and homo/lesbophobic father if she disclosed that she was lesbian:

**Table:** 

Lab	Clause
CA	*Marcela: “(…) He [Marcela’s father] always said that if he had a gay or lesbian child, or black, or whatever, he killed him*,
RE	*then I had to hide, to hide, to hide.*
CA	*So what I did then was to date men…” (N1, EP 1; L 8:10)*
RE	*“I mean I tried to convince myself I was straight, ‘I’m straight, I’m straight, I’m straight’*,
EV	*to avoid problems, because my dad was so strict, and also was violent…” (N1, EP 1, L13:14)*

Thus, participants’ narrative revealed the intense family pressures to conform to a heterosexual path, even when they were aware of their same-gender feelings. Participants’ heteronormative family pressures were visible in all participants’ accounts and they did not appear to question whether their parents were right to apply this pressure. Also, the lesbophobic family contexts were mainly portrayed as the main reason to avoid identifying as a lesbian by those participants who were aware of their same-gender feeling before becoming mothers.

#### Building a Relationship and a Family With a Man

Since participants considered that lesbianism was not an option for them, they all built relationships with men or tried to follow a socially expected heterosexual path. Furthermore, all participants tried to build a family with their child/children’s father when they became mothers. While three participants (Camila, Paula and Jimena) had planned their first pregnancy, the other five (Teresa, Julia, Carla, Marcela and Beatriz) were not expecting to become mothers when they did. Paula, who had planned her pregnancy, narrated an episode that illustrated her desire to be a mother. In addition, Paula’s account showed that she initially felt attracted to her children’s father, as indicated in the narratives of Teresa and Julia also. However, Paula said that she had planned to form a *“conventional family”* with her male partner in order to avoid being discriminated against. Paula was aware of her lesbian desire, yet the expected rejection and the anticipated “suffering” of living out a lesbian identity was seen as risky for her. Paula’s account revealed the minority stress she experienced as a young lesbian living in Chilean society, thus, heterosexual marriage was seen by Paula as a much safer place:

**Table:** 

Lab	Clause
AB	*Paula: “I was interested in making a family*,
OR	*I wanted to be a mom, have children and it would be difficult with a woman*,
CA	*so I met my future husband, and I said ‘wow’ I felt in love at that time…” (N1, EP 2, L 168:170)*
OR	*“I was 18, then I said actually, between having a relationship with a guy who I’m going to marry*,
EV	*I will have the expected family, the conventional family; or taking the risk in the life and suffering*,
EV	*having problems, because they might not understand me, they will discriminate me, they will reject me;*
RE	*I prefer to marry him, then I married…” (N1, EP 2, L 175:178)*

Like Paula at a similar point in her life course trajectory Beatriz, who was from another southern and traditional city, Rancagua, also opted for having a heterosexual relationship instead of expressing her lesbian feelings. Beatriz had a short lesbian relationship when she was younger, but Beatriz thought that God would “punish” her because of her forbidden non-heteronormative behaviour. The internalised lesbophobia experienced by Beatriz revealed the strong impact of religious discourses about prescriptive heterosexuality upon her identity formation. Thus, Beatriz’s prior religious beliefs played a crucial role in her decision to pursue a heterosexual relationship. Although Beatriz had not planned her pregnancy, she had thought previously about the idea of having a child. Then, when Beatriz met her daughters’ father, she saw him as the prospective parent for her future children:

**Table:** 

Lab	Clause
OR	*Beatriz: “Before, I had had a relationship with this girl, as I told you, I was around fifteen…*
EV	*I thought I had taken the wrong way, thinking that maybe God punishes you.*
CA	*Then I decided to take the right course, and he [the daughters’ father] had been interested in me during the summer*,
EV	*so I decided to accept, I liked him*,
EV	*I had thought he could be sometime the father of my children, as I said to you*,
RE	*then we started a relationship and we agreed on many things…” (N1, EP 1-2, L 382:388)*

Family heteronormative expectations pressured participants to have a relationship with a man and to get married in some cases. As participants’ narrative revealed, lesbianism was associated with suffering for some interviewees and punishment for those who held Christian religious beliefs.

### Experiencing a Lesbian Desire That Needs to Be Expressed

Participants’ narratives revealed a renewed period in their lives during which they started to rethink their attraction to women and considered that identifying as a lesbian was the best life course identity project for them. Simultaneously, participants started to question the relationship they had with their child/children’s father. For all participants, this process started during their trajectory as a mother. This theme was fed by two sub-themes: “*Rethinking lesbianism as a life course identity project”* and *“Questioning the heterosexual family life project.*”

#### Rethinking Lesbianism as a Life Course Identity Project

During this time, participants began to view lesbianism as an available option for them and they then affirmed their lesbian identity. The three participants who had previously not identified a clear lesbian desire (Camila, Julia and Jimena) started to recognise their same-sex feelings as a stable feeling of attraction during this time. Camila previously had considered that having a relationship with a woman was not a possibility for her. Camila said that lesbians were not visible in Chilean society, therefore she had not represented in her mind what being a lesbian was. Camila narrated an episode to portray how and when she started to realise that lesbianism was an option for her and how she then finished her relationship with her daughter’s father. As Camila’s account unfolded, media representations of lesbian identities made a clear difference in helping her to recognise her lesbian feelings:

**Table:** 

Lab	Clause
CA	*Camila: “and some pictures of lesbian couples were shown on TV [She was watching TV with her daughter]*,
OR	*I have never thought in my life that this existed…*
EV	*within my little world at that time it wasn’t an option (…)*
EV	*so I stayed like with the doubt, and the doubt began to grow as more, more, and more*,
EV	*and I began to find out more, more and more, until I realised there were many lesbian series [on Internet]… (N1, EP 2, L 104:109)*
RE	*And a world began to be open to me*,
CA	*and at some point I said to my daughter’s father, ‘You know what, like something…’*
EV	*I didn’t know what it was yet*,
RE	*but I told him ‘I need time to be alone…’” (N1, EP 2, L 123:130)*

The five participants who previously had realised they had attractions to other women during their adolescent years (Teresa, Carla, Paula, Marcela, and Beatriz) began to re-examine their same-gender feelings during early motherhood. During this time, these participants became aware of the prominence of their lesbian desires and began to realise that they did not feel attracted to their male partner. The following episode within Teresa’s account clearly illustrated the prominence of her erotic attraction to women and how she then began to affirm her lesbian identity:

**Table:** 

Lab	Clause
EV	*Teresa: “Before I felt it was normal, that was a normal process, that I could like men*,
EV	*but it was like fool me, because basically I was super clear that I didn’t like men at all.*
EV	*Not now, if you ask me, I feel women are the only things that move me, I don’t like men at all…” (N2, EP 1, L 328: 330)*
EV	*“I began to realise, it so funny*,
CA	*when I went to the gym and there was a teacher of gymnastics*,
OR	*the teacher of cardio kickboxing who I loved*,
EV	*so I said ‘Ok, I love her’, but because I really loved her, you know*,
EV	*I mean, it wasn’t that I liked her because I found her cute, pretty, no*,
EV	*the girl shook all my hormones, I don’t know, but I really loved her…” (N2, EP 1, L 354: 359)*
EV	*“Then I felt I was going into really heavy things*,
RE	*and by 2010, I definitely saw myself as lesbian…” (N2, EP 1, L 367: 368)*

It is important to note that during this life course period, all participants (except Marcela) developed positive views about their own lesbian feelings. This contrasted with the mainly heteronormative expectations from their parents.

#### Questioning the Heterosexual Family Life Project

As noted above, during this period of growing awareness of their own lesbian feelings participants also noted their lack of attraction to their male partners, which had evaporated in those who previously had felt attracted to them. Consequently, participants started to question the heterosexual path they had trodden previously. However, breaking the heterosexual relationship they had built with the father of their child(ren) entailed a significant challenge for them as participants had a joint home with their male partner and had formed a family based upon it. Finishing the heterosexual relationship they saw as bringing the “destruction” of their heterosexual family life project. In spite of these challenges, seven participants had finished their relationship with their child(ren)’s father by the time they were interviewed for this study and had opted for having a relationship with a woman. Again, parental heteronormative expectations were apparent in participants’ life course stories. In some cases, parents’ religious beliefs portrayed heterosexual marriage as the desired goal for participants as Christian daughters. For example, in Carla’s account, heterosexual marriage was seen as a representation of “happiness” for her Christian parents. The narrative piece below revealed Carla’s varied attempts to maintain her heterosexual marriage over several years and the influence of Carla’s own mother’s and father’s religious expectations on Carla’s effort to do this:

**Table:** 

Lab	Clause
OR	*Carla: “I used to do everything [because Carla’s husband did not have a job], but I was persistent and I said ‘no, it has to work’*
EV	*because my mom was happy, because my dad was happy*,
EV	*because I had already made the decision to form a [heterosexual] family.*
EV	*I think that was very important for them*,
EV	*I mean, my mom always had told me that she was happy to see me get dressed in white to the church*,
CA	*and I say her ‘no mom, that’s not gonna happen’*
EV	*and I tried, I tried to be with him for 4 years, but no, I couldn’t, I couldn’t*,
CA	*and then in 2009 I made the decision, I said to him [her husband] ‘you know what, this will not work’…” (N1, EP 3, L 39: 46)*

In contrast to the rest of the participants, Marcela, had not finished her heterosexual relationship at the time of the interview with her. Nonetheless, she had been in a lesbian relationship for about 5 years before she was interviewed. Although, Marcela’s lesbian desire a prominent feature in her mind, she still believed that having a father and a mother was the best option for her daughter and said that she could put up with her heterosexual marriage for this. Interestingly, as with Carla’s account above, Marcela’s account illustrated the influence of her mother and also Marcela’s husband who put emotional pressure on Marcela’s decision making. Marcela’s own daughter also put pressure on Marcela not to leave and end the parental couple relationship. Marcela’s own Christian religious beliefs and values made her feel guilty and scared because of God’s expected punishment. Marcela’s mother also avoided talking about Marcela’s lesbian feelings. It seems that some participants’ mothers (and also fathers) used silencing as a strategy to avoid acknowledging their daughter’s feelings making lesbianism something apparently non-existent with the effect that lesbian desire and happiness was rendered as an insufficient reason for dismantling the heteronormative family:

**Table:** 

Lab	Clause
CA	*Marcela: I tell her ‘Mom I want to talk to you’ and she says ‘Oh, no’ and she leaves*,
EV	*but she realises, but she prefers to look like silly, she doesn’t want to take it, she doesn’t want to assume it, she will not assume it” (N2, EP 3, L 50-52)*
CA	*“Once I told him [her husband] that I wanted to leave home, but I didn’t explain why to him*
CA	*so he said ‘how are you going to do that to your daughter?, Remember that you suffered when you were a child*
CA	*and I don’t think you want the same for her’*
CA	*and my daughter says, ‘I don’t want that you to leave my dad’*
EV	*So I have too many family pressures, and I can’t live my condition openly” (N2, EP 3, L 68-72)*
RE	*“And I always ask God for forgiveness [because of her lesbian relationship]*,
EV	*I am scared that God punish me” (N2, EP 3, L 355-356)*
CA	*“I ask God: ‘please make the love I feel for her, the love for my husband”’ (N2, EP 3, L 405)*

As mentioned above, family pressures and expectations were seen at their strongest when participants started to think about leaving their male partner and break apart the heterosexual family project they had built. This process had taken years to do for participants who had separated from their male partners at the time of the interviews. In addition, when participants thought about expressing their lesbian desire, religious expectations were meaningful for those who held Christian beliefs and/or had Christian parents. Religious expectations were particularly prominent in Marcela’s and Beatriz’s narratives, the participants who came from Chilean regions other than Santiago.

### Conveying Sexual Identity to Family of Origin, Friends, and the Child(ren)’s Father

Conveying sexual identity to others was a relevant aspect of participants’ coming out narratives. Nevertheless, participants varied in the level of their disclosure to others. Despite individual variations in participants’ coming out to others, the findings of this study revealed more instances of disclosure to people who were emotionally close to them like their family of origin and their close friends than to people who were distant. Although only a minority of participants had disclosed to their child(ren)’s father, the challenges participants encountered during this particular disclosure process were an important aspect within these participants’ narrative accounts. This theme split into three related but distinctive sub-themes: “*Negotiating lesbian identity with family of origin;” “Negotiating lesbian identity with friends;”* and *“Negotiating lesbian identity with the child(ren)’s father.*”

#### Negotiating Lesbian Identity With Family of Origin

While four participants mentioned that they had disclosed to at least one member of their family of origin, the other four interviewees reported that they had not disclosed to any of their family of origin members at the time of their interview. Nevertheless, three of the participants who had not disclosed to their family members thought that their parents had realised the participant’s attraction to women in other ways.

All participants noted the importance of family support in their lives, or emphasised their desire to be accepted by family members, mainly by their parents. Disclosing participants reported at least one family member who was accepting of the participant’s lesbian identity. Julia’s main story conveyed how important the acceptance of her mother and grandparents was to her. She implied that their acceptance had improved over time after Julia’s parents’ initial adverse reaction to Julia’s coming out. Julia’s narrative further revealed her grandparents’ beliefs that being a lesbian would be incompatible with Julia having more children and how much the lens of reproductive motherhood influenced family members’ thoughts. In contrast to her careful focus on coming out to her family of origin, Julia only very briefly mentioned coming out to her friends and how she felt supported by them:

**Table:** 

Lab	Clause
AB	*Julia: “I came out publically right away*,
CD	*I mean, not publically, I didn’t publish anywhere*,
CA	*but I told my family, I told my loved ones*,
CA	*and I told them that they had to accept me how I was…” (N 2, EP 1, L 29:32)*
EV	*Julia: “And I feel supported by the people that love me, my family, my friends…” (N 2, EP 1, L 71)*
CA	*Julia: “In the beginning it was hard for my mom, but after she realised it wasn’t an issue for her.*
EV	*It was more difficult for my grandparents.*
CA	*They asked me if I would have more children*,
EV	*and I told them yes, that I could have more children. That it didn’t mean that*,
CD	*Then, they relaxed. At the moment, it’s not an issue [for Julia’s parents and grandparents]…” (N 2, EP 1, L 471:475)*

Camila, who also had disclosed to her family, still did not feel properly accepted by her mother. Camila had disclosed to her mother after she met her first lesbian partner. Her narrative showed how her mother had accepted neither the lesbian partnership nor Camila’s lesbian identity since then. Camila’s account also indicated her mother’s traditional expectations of a married woman’s role in the home and her mother’s close interest (and policing) of this. Camila used the metaphor *“se le cayó el pelo”* (*“her hair fell out”*) to portray how disappointed her mother felt about her lesbianism and Camila’s transgression of the conventional gender norms. After the disclosure Camila’s mother continued to attempt to silence Camila’s lesbian identity by letting her disapproval and shame be known but not open for further discussion, as Camila explained with the evocative phrase in Spanish *“llorando por los rincones”* (*“crying in corners”*):

**Table:** 

Lab	Clause
AB	*Camila: “I told her [her mother] after 1 month I met Antonia [her first lesbian partner]*,
CA	*because my mom realised ‘so what’s up? Why are you going out a lot, you haven’t done that before’*
CA	*’ok, I’m dating someone’ I told her*,
CA	*and my mom was so disgusted*,
CA	*but how you are dating someone? you, a woman, a married woman, that loves her home’ according to her*,
CA	*and I said ‘she is a woman’*
CA	*and then her hair fell out (laughs)” (N 2, EP 3, L 268:276)*
CD	*“Long time, I think she is still crying in the corners.*
CD	*Camila: Still nothing, nothing regarding the issue [her mother had not accepted Camila’s lesbian identity]…” (N 2, EP 3, L 284)*

Thus, participants found positive and negative reactions after their coming out to their parents. However, family heteronormativity and the rejection of lesbianism were mentioned by most participants. Sometimes these heteronormative expectations were expressed through criticism and emotional pain (“suffering,” “crying,” “disgust,” disappointment and hopelessness). Nonetheless, participants’ parents (particularly mothers) usually continued providing support for participants’ parenting as Camila said *“I used to leave her (Camila’s daughter) at my mom’s home after school” (L 595) “My mom is always there, supporting me” (L 884).*

#### Negotiating Lesbian Identity With Friends

As was noted above in relation to Julia’s account, disclosure to and acceptance by friends was a significant aspect within participants’ sexual identity life course but this did not receive the same prolonged painstaking focus that participants gave to describing maternal and paternal reactions. All participants reported that they felt accepted by at least one friend. Participants’ friends’ reactions contrasted with their family of origin’s mainly negative reaction after coming out. Acceptance from friends was important for participants’ lesbian identity affirmation within the lesbophobic context in which they lived. Carla’s account illustrated how she had openly expressed her lesbianism with her closest friends. Carla used a Spanish equivalent of Weston’s (1991) phrase “family of choice” to describe her friends as *“the family one chooses*” *(“la familia que yo escogí”)* to portray the importance these emotional ties had for her:

**Table:** 

Lab	Clause
OR	*Carla: “but, for example, I have a group of friends, the friends of my life.*
OR	*We’ve been friends for about 20 years, since we were classmates*
CA	*And interestingly we were all gay*,
CA	*at that time nobody knew (…)” (N 2, EP 4, L 208:211)*
EV	*“And they all love my daughter, and my daughter loves them” (N 2, EP 4, L 214)*
CA	*“Actually, I must say that only my family, the closest one [her closest family members], don’t know*,
EV	*the family I chose, who are my friends, they all know…” (N 2, EP 4, L 218:219)*

#### Negotiating Lesbian Identity With the Child(ren)’s Father

In contrast to their disclosure to family of origin and friends, at the time of interview only one participant had chosen to disclose to her child’s father. Another four participants had been confronted by their ex-male partners to acknowledge their sexual identity because their children’s father previously had begun to think that the participant might be attracted to other women. Thus, at the time of the interview, participants were often negotiating either how to convey or to conceal their sexual identity from their child’s father.

Participants struggled when tried to convey their sexual identity to their child(ren)’s father. Within participants’ life course narratives, these were the clearest example of lesbophobia and patriarchal attempts to subordination that were appreciated as such by participants themselves. In fact, the five participants whose their ex-male partner had acknowledged the participant’s lesbian identity reported only encountering negative reactions from them. Camila, who had disclosed to her ex-male partner when he realised Camila was repeatedly meeting her first lesbian partner, described her daughter’s father’s negative reaction to Camila’s attraction to women. Prior to this, Camila’s daughter’s father had expressed no concerns when Camila first dated a woman but perhaps the persistence of Camila’s commitment to dating women emphasised to him that Camila was not going to go back to her previous relationship with him:

**Table:** 

Lab	Clause
AB	*Camila: “I had my first partner*,
AB	*and he obviously realised…*
CD	*but he… I think it’s a problem for him until today*,
EV	*I think it must have been so strong for him…*
CA	*and he said ‘ok, but do not worry, I am so open-minded, it doesn’t matter for me.’ (N2, EP 2, L 222-226)*
CA	*Camila: “The thing is that I told him… he asked me what I was doing in my life*,
CA	*and I told him ‘ok, I’m dating a girl’ [another partner]*
EV	*and then I remember that if he was been able to overturn the table*
EV	*with the juices we were drinking*,
EV	*I think he would have taken them [the juices] and thrown them like… so angry, with a face of rage*,
CA	*and I told him, ‘but what’s up with you?… but if you know, you know that I like women, and I will continue to like them’*,
CA	*he told me ‘no’, and he was angry” (N2, EP 2, L 324-331)*

Three of the four participants, who had not disclosed, had hidden or denied their lesbian identity from their ex-male partner to avoid any possibility of losing the custody of their child(ren). Participants feared being manipulated or controlled by their ex-male partners as they thought they were in disadvantaged position within the Chilean lesbophobic legal context. Paula’s story showed the fears Paula had about her children being taken away because she was a lesbian and how the children’s father would be able to exercise his will and power in Chilean courts. Paula mentioned the case of Karen Atala (who lost custody of her children as mentioned above) to convey how restricted she felt in her local social context:

**Table:** 

Lab	Clause
OR	*Paula: “My ex-husband didn’t know about my inclination*,
CA	*and I always feared that he could realise and take away my children.*
EV	*At that time the case of Karen Atala was well known, then I lived with a great fear*,
RE	*so I had to live a double life” (N2, EP 2, L 22-25)*

### Conveying Maternal Sexual Identity to the Children

As participants recognised their need to express their lesbianism, and from this began to build a lesbian relationship, they started to re-think the way they conveyed their identity to their children. Participants’ children had all been born in the context of a heterosexual, family, therefore participants needed to reformulate many aspects of themselves and their stories in order to come out to their children. This theme was fed by two sub-themes: “*Avoiding the disclosure of lesbian relationships to the children”* as often participants initially attempted to avoid disclosure only to find that they later began *“Preparing the child for coming out as a lesbian mother.”*

#### Avoiding the Disclosure of Lesbian Relationships to the Children

Participants displayed two main strategies in concealing their same-gender relationships from their children. Firstly, each participant initially avoided disclosing her sexual identity to their children. Participants in all cases said they had subsequently presented their first female partner to their children as a “friend” in order to conceal their own sexual identity. It seems that within the Chilean lesbophobic context in which participants lived, being an open lesbian mother was not a possibility for them initially. Camila narrated an episode that showed how she presented her lesbian partner as friend to her daughter even when the three of them started to live together. Her account also illustrated how presenting her partner as friend necessitated Camila avoiding receiving or expressing affection from or to her partner:

**Table:** 

Lab	Clause
CA	*Camila: “and at some point, I didn’t tell Fran [Camila’s daughter] about it [that Camila was living with a female partner]*,
CA	*it was like ‘Marce is my friend, we sleep together, but she’s my friend’…’*
OR	*The flat had two bedrooms, one for the child and one for us, like now*,
CA	*and it wasn’t like telling Fran, “look Francisca, Marce is my partner’ I’m a lesbian.”(…)*
EV	*But Fran, [was]a girl that after all was 2 or 3 years younger, some things [she] could understand and others things do not…*
EV	*we weren’t affectionate between us in front of Fran, for the same reason, to avoid any conflict…” (N4, EP 2, L 609:615)*

Secondly, it followed on from non-disclosure that some participants tried to hide their lesbian affective expressions, as was noted above in Camila’s narrative. This strategy was closely associated with presenting a lesbian partner as “a friend.” Both strategies contributed to concealing a participant’s sexual identity from her children. Four participants (Teresa, Camila, Julia and Paula) explicitly reported attempts to hide lesbian affective expressions. Julia’s account revealed why she opted to hide affectionate expressions for her partner in front of her daughter: Julia had received this advice from the psychiatrist who she had been to see with her daughter’s father. The following narrative passage illustrated how Julia conformed to conceal her affectionate expressions for her partner at this point in her life, mainly from Julia’s continued concern to respect her ex-husband’s wishes as in effect voiced by the professionals they had seen:

**Table:** 

Lab	Clause
EV	*Julia: “I found her [the psychiatrist] very prohibitive*,
EV	*like everything was abnormal, like I couldn’t hold her hand [partner’s hand] or*
EV	*I couldn’t make visible any affection with my partner…” (N2, EP 3, L 157: 159)*
CA	*“she told me that we couldn’t go to the beach together or*
CA	*that we couldn’t sleep together, things like this…*
EV	*like prohibitive and restrictive” (N2, EP 3, L 165: 167)*
RE	*“I respected what she said anyway, because I went with my daughter’s dad*,
EV	*and it has been very important to go to an specialist with him, either a psychiatrist or a psychologist…” (N2, EP 3, L 170: 171)*

Julia’s account also revealed that lesbianism was seen as something abnormal by others, in her case by a psychiatrist and that some family members were prepared to seek and also receive societal endorsement to enforce a heteronormative picture even if this was a facade. The pathologisation of homosexuality was a prejudice visible in all participants’ accounts. As Julia did, other participants also heard that lesbian affectionate expressions were something inappropriate for children to see or hear about. Concealing any presentation of their lesbian identity from children also continued to fuel participants’ concerns upon their own uncomfortable feelings about their lesbian identity. Thus, heteronormative expectations also pressured participants to avoid expressing their lesbian feelings or disclosing to their children.

#### Preparing the Child for Coming Out as a Lesbian Mother

Despite the pressure to conceal disclosure to their children remained a significant goal of participants’ sexual identity life courses for reasons of authenticity but participants deemed it important prepare their children for any disclosure. One preparation strategy was the teaching of tolerance to their children (against the lesbophobic context). This strategy was identified in the narratives of six participants (Teresa, Julia, Paula, Jimena, Marcela, and Beatriz). Julia portrayed the strategy of teaching tolerance in her micro story concerning how she talked to her daughter in order to prepare her for disclosure:

**Table:** 

Lab	Clause
AB	*Julia: “No, I don’t [she had not disclosed], but I read tales to her every night*,
CA	*and many times I tell tales where tolerance is essential*,
EV	*tales that show family diversity, the rainbow and things like that*,
RE	*to make her know that she has to tolerate everyone, an Asian, a black person, an homosexual, anyone…” (N2, EP 3, L 127: 131)*
EV	*“I hope this [the disclosure] be as normal as possible for her [her child], the most natural thing*,
EV	*I want her to grow up with the tolerance impregnated in the blood…(”impregnada en la sangre” in Spanish)” (N2, EP 3, L 508: 509)*

Although only three participants (Camila, Paula, and Beatriz) had disclosed to their children at the time of their interviews, as other four interviewees (Teresa, Julia, Carla, and Jimena) planned to do this later. Only Marcela, still married, had not planned disclosing to her daughter because she feared negative consequences, as noted previously.

Paula narrated an episode about the time when she disclosed to her two sons and her daughter and how she had felt accepted by them. In the same narrative piece, Paula reported how previously she had felt fearful about the possibility of being rejected by her children. In particular, Paula noted that she felt afraid of her daughter’s possible reaction, revealing Paula’s ideas about gender impacting upon children’s reactions her sexual identity disclosure. Paula carefully began her account by saying that she had prepared the children for “at least 2 years” before she told each of them individually. She implied that the lesbophobic context in which she and her children lived made it difficult for her in coming out as a lesbian mother:

**Table:** 

Lab	Clause
EV	*Paula: “after many questions, I think at least for about 2 years*,
EV	*thinking about how telling them, and putting myself in the worst scenario of thinking how they would react*,
EV	*because although I had raised them alone, there is a social pressure, there are [sexual] prejudices that surround us*,
EV	*in the context, in the school, among friends, in the family, etc.*
EV	*you always have the fear of how they [the children] would react.*
EV	*One of those fears, the main was to be rejected by them, that they didn’t love me*,
EV	*(…) in particular my daughter, that she didn’t want to be touched by me… I was very afraid” (N2, EP 2, L 40:46)*
CA	*”However, when I decided to talk to each of them, I talked with them alone, I mean with each one*,
EV	*their response was amazing [because she felt accepted by them]…” (N3, EP 2, L 48:49)*

The disclosure to children was a challenging life course goal for participants, but not because of the participants’ own transition to lesbian identity but the context of external prejudice. Participants thought that within Chilean society, a lesbian was not a good model for children, and feared that others’ prejudices could impact their children’s wellbeing.

## Discussion

The purpose of this study was to explore how sexual identity and motherhood were negotiated in the private and the public domains. We investigated the life course experiences of a group of Chilean LM who had conceived their children through a previous heterosexual relationship. We found that traditional family values and Christian religious beliefs played a significant role in the narrated stories of LM in Chile and that these were powerfully displayed by family of origin members when resisting their daughter’s journey into lesbian motherhood yet family of origin mothers continued to play an important role in supporting their daughter’s parenthood.

We found that Chilean LM undertook a long journey to reconcile with their own identities, and this was particularly hard for those LM who defined themselves as Catholic. Interestingly, the impact of the lesbian mother’s own religious beliefs and values (or those held by her family of origin) on their own internalised lesbophobia appeared noticeably more pronounced among lesbian mothers from regions outside Santiago. This is a particular aspect of LM identity life course had not been described in previous studies with LM living in Latino countries, although Tuthill (2016) has identified a similar patterning among Catholic Hispanic LM living in the United States.

Another particular aspect of Chilean LM in the present study appeared to be the impact of Karen Atala’s judicial case on some participants’ fears of losing the custody of their children, something that has not been described in other studies with LM living in Latino countries. We neither observed that participants in our study lived in constant fear of being attacked as studies with Brazilian lesbian mothers have indicated (Pinheiro, 2006; Santos and Alves de Toledo, 2006) nor noted that Chilean lesbian mothers focused upon directing their children to avoid showing signs of affection for same-gender peers unlike Mexican lesbian mothers (Haces, 2006). Nevertheless, some participants in our study had feared physical violence on occasions or expressed some concern about how their child’s behaviour might be interpreted by others. Our qualitative investigation, with a small non-randomly drawn sample, certainly precludes a definitive pronouncement regarding cross-cultural comparisons of lesbian motherhood.

Participants who had realised about their attraction to women during adolescent years considered this attraction as inconsistent with the socially expected heterosexuality as noted in other studies (Asencio, 2009; Jara and Araujo, 2011; Palma et al., 2012). Some of our participants tried to hide or deny their attraction to women to their parents (see also Acosta, 2010) because they feared the consequences of being seen as a lesbian by them. Participants often associated same-gender attraction with anticipated “suffering,” “punishment,” and “rejection” which reflected the minority stress they experienced (Meyer, 2003). In our sample, participants’ parents’ heteronormative expectations and Christian beliefs and values underlay parental pressures. The association of heteronormativity with parental Christian religiosity in the socialisation of Latina lesbians also had been previously described by research conducted in the United States (Espín, 1987; Acosta, 2008, 2010; Asencio, 2009; Tuthill, 2016) and Chile (Herrera, 2007; Jara and Araujo, 2011).

All participants had become pregnant through having a heterosexual relationship. Then, the arrival of a child represented for most participants their reason for continuing the relationship with their male partner. Furthermore, participants’ parents’ expectations of heterosexual family formation, further supported participants’ attempts to maintain a “conventional” family. In a study with Puerto Rican lesbian migrants to the United States, Asencio (2009) also found that lesbians felt constrained by their family’s expectations to get married to a man and have children. Our study in a Chilean context has additionally emphasised how parental pressure can keep lesbians in a heterosexual marriage years after realising their love for another woman. This reflects how difficult it was for this group of Chilean LM to identify as a lesbian at that time, when the Chilean socio-legal context was considerably more restrictive than today.

Behind the themes in our data could be seen terrifying glimpses of Latino heteropatriarchal violence (Marcela’s father and Camila ex-male partner) and psychiatric oppression. Breaking the heterosexual relationship that they had previously built with their child(ren)’s father entailed significant challenges for participants. Those participants whose ex-male partner had acknowledged the participant’s lesbian identity reported only encountering adverse reactions from them. Further, finishing the heterosexual relationship brought the “destruction” of the heterosexual family life project they had built and also challenged the social approval of their parents. Thus, the process of separation was a long and painful process for most participants. Only two participants separated shortly after they acknowledged their lesbianism.

Another important goal for participants in this study was disclosing to significant others in their lives: their closest friends and their family of origin, in particular, their own parents. While all participants had disclosed to friends and had felt accepted by at least one of them, only half of the participants had disclosed to their parents. Participants’ narratives revealed that close friends were important for their own acceptance and lesbian identity affirmation and in the main they had encountered positive reactions from their friends which might have emboldened them. While some participants had received emotional support from heterosexual and non-heterosexual friends, others only had been open or had felt accepted by non-heterosexual gay or lesbian friends. One participant highlighted the importance of the emotional support she received from her non-heterosexual friends by describing them as *“the family one chooses”* or the “family of choice” as has been widely described in anthropological or sociological research on lesbian and gay families (Weston, 1991).

In contrast with participants’ friends’ mainly positive reactions, participants’ parents reacted in diverse ways. Of the four participants who had disclosed the sexual identity to their parents, three had felt in some respects still accepted by their parents (Lynch and Murray, 2000; Santos and Alves de Toledo, 2006). The other participant who had disclosed reported that her mother still rejected her lesbianism (Espín, 1987; Sánchez et al., 2004; Acosta, 2008, 2010; Asencio, 2009). Similarly, of those participants who had not deliberately disclosed their sexual orientation to their parents, three had encountered adverse reactions when their parents realised in other ways that their daughter was a lesbian. These participants stated that mothers were more active in stating their rejection of participants’ lesbianism than were fathers. One of the main strategies used by participants’ mothers was to avoid talking about, or acknowledging, any aspect of lesbian life with participants, yet participants knew that their mother was deeply upset and ashamed of their lesbian daughter or as Camila vividly described her mother as “crying in [the] corners” about it. Participants considered that their mothers used this strategy to try to render lesbianism as something that did not exist, did not happen, or make it invisible – to not mention it and carry on as normal. Acosta (2008), in her study with Latina lesbians, also found that some families tried to erase non-heterosexuality by using control and manipulation tactics. Interestingly, the four participants whose parents had rejected their lesbianism conveyed that religious values were held by their family of origin and that these were associated their family objections (see also Acosta, 2010; Jara and Araujo, 2011).

Despite parents’ negative attitudes toward their daughter’s lesbianism, all participants were still in contact with their family of origin and had received emotional support from their parents in other respects (Lynch and Murray, 2000; Sánchez et al., 2004; Jara and Araujo, 2011). Some participants also continued to receive help for childrearing or economic support from their parents. Even though most participants lived independently from their parents they continued to live close by, and their lives were intertwined (Lynch and Murray, 2000; Swainson and Tasker, 2005). For example, Camila’s mother, who was now silent on her daughter’s lesbianism, was regularly helping Camila out with childcare. Thus, parents continued to be an essential source of support for participants and this continued support probably weighed heavily in participants continued thinking about how to live as a lesbian mother. Previous studies with Latina lesbians also found that Latino families do not necessarily expel their daughter from the family circle (Espín, 1987; Acosta, 2008, 2010; Asencio, 2009) and the finding from our study give further insight into the complex working of acceptance and support in Latino families. The strong support participants received from their family of origin suggest that closeness and loyalty of Latino families, often described as familismo, possibly contributed to the wellbeing of participants despite the level of minority stress they experienced from their families (Ayón et al., 2010).

Participants initially avoided disclosing her sexual identity to their children and then displayed different strategies in conveying their same-gender relationships to their children. Thus, participants had introduced their first female partner to their children as a “friend” and then avoided any demonstration of affection with their partner. However, some of our participants were teaching tolerance to their children with a view to preparing them for disclosure (Mitchell, 1998; Gartrell et al., 2000; Jara and Araujo, 2011). Again, our findings here are similar to findings in Jara and Araujo’s (2011) study in which some Chilean LM initially presented their lesbian partner as a friend to their children. Research studies conducted with Latina lesbians (Acosta, 2010) and LM (Palma et al., 2012) also have revealed that lesbians usually presented their same-gender partner as friends to their families. Those participants who had disclosed their sexual identity to their children, reported having felt accepted by them (Jara and Araujo, 2011). Some participants had decided to delay disclosure to their children following the advice of friends, their own beliefs regarding non-heterosexual disclosure, or the advice of a therapist.

Following a life course theoretical analysis (Allen and Henderson, 2016), it seemed that despite participants’ first attempts to conceal or hide their sexual orientation, most of them were able to subvert social forces that constrained them in identifying as a lesbian while being a mother. This indicates the crucial role of human agency (Allen and Henderson, 2016) in enabling participants to choose for their own life course pathway despite the lesbophobic and restrictive legal context in which they lived (Babbitt, 2013). Additionally, it seemed that acceptance from significant others, such friends and their own children, and latterly parental (mainly maternal) support helped to participants’ own self-acceptance and identity affirmation, revealing the powerful weight of interdependence and linked lives in shaping a lesbian identity pathway.

### Strengths, Limitations, and Recommendations

This study provided an insightful understanding of the family life of Chilean LM post heterosexual relationship dissolution. A case-centred (Riessman, 2010) or the idiographic methodological approach contributed to this accomplishment as well as the careful selection of a homogeneous sample within which to explore a distinctive range of factors. As Smith and Osborn (2008) have noted, purposive and homogeneous sampling can allow for a detailed examination of participants’ accounts.

The findings of this study might have a limited generalisability and might be less applicable for Chilean LM who do not fit with features of the samples selected. Based on this limitation, it would be worth conducting research with LM who have children in the context of a lesbian relationship, and those who identify as working-class women. It may also be relevant to examine the experiences of children of Chilean LM and the extent to which they are exposed to discrimination and how such negative social forces might impact on children’s well-being.

The findings of this study also represented the narratives of lesbian motherhood within a particular socio-historical time and context. Thus, future generations of LM might encounter different experiences over their life courses by navigating in changing social contexts. The Chilean legal context is changing, and it is more supportive of LGBTQ people than at the time of the interview. Indeed, same-sex civil parentship has already been approved in Chile after our study was conducted. However, same-gender couples still encounter many legal restrictions in Chile. Additionally, narratives of lesbian motherhood might be substantially different in other Latino countries. Yet, considering the impact of heteronormativity and religious beliefs and values on the family life of Chilean LM, the findings of this study might also be applicable in Latino countries or indeed elsewhere when a strong influence of Christian Churches on gender, sexual and family values exists.

Clinical psychologists and social workers working with LG parents should help LM to become aware of the impact of internalised lesbophobia on their family decisions, plans, and/or expectations. Social and/or emotional support might help LM to cope with enacted or subtle forms of sexual stigma presented in Chilean society. Children of LM might also benefit if their mothers acknowledge the importance of family and community support in coping with an oppressive social context. It would also be worthwhile to train school teachers or health care providers about the impact of either discrimination or support on the family life of LM and their children.

## Conclusion

The findings of this study have revealed the strong impact of lesbophobia on the life course experiences of LM living in Chilean society. Enacted forms of sexual stigma (Herek et al., 2009) seemed to be marginal in participants’ narratives. However, the lesbophobic context exerted a substantial influence on what LM felt they could reasonably do, revealing how participants had internalised the lesbophobia from Chilean society. Furthermore, anticipated discrimination of their children seemed to be a major concern for LM in this study. Nevertheless, despite LM’s concerns relating to the anticipated discrimination of their children, no participant in this study reported that their child had encountered experiences of discrimination. Additionally, all the participants reported at least one experience of acceptance by a significant other such as a family member or friend, revealing the polarisation of participants’ experiences and/or expectations, and the tensions between oppressive and supportive social forces.

## Data Availability Statement

The datasets presented in this article are not readily available because participants signed a consent protecting their privacy, and only pseudonymized extracts of transcriptions were authorised to be published. Requests to access the datasets should be directed to victor.figueroa@uniacc.cl.

## Ethics Statement

The studies involving human participants were reviewed and approved by the Departmental Ethics Committee, Department of Psychological Sciences, Birkbeck University of London. The patients/participants provided their written informed consent to participate in this study.

## Author Contributions

VF contributed the literature review, data collection, and analysis. Both authors contributed equally to the design of the work, the drafting, and revising of the manuscript.

## Conflict of Interest

The authors declare that the research was conducted in the absence of any commercial or financial relationships that could be construed as a potential conflict of interest.

## Footnotes

1 The present studied was conducted before the approval of the Civil Union. Thus, at the time of the interviews, the Chilean socio-legal context was more restrictive than nowadays.
